# Automated stenosis estimation of coronary angiographies using end-to-end learning

**DOI:** 10.1007/s10554-025-03324-x

**Published:** 2025-01-09

**Authors:** Christian Kim Eschen, Karina Banasik, Anders Bjorholm Dahl, Piotr Jaroslaw Chmura, Peter Bruun-Rasmussen, Frants Pedersen, Lars Køber, Thomas Engstrøm, Morten Bøttcher, Simon Winther, Alex Hørby Christensen, Henning Bundgaard, Søren Brunak

**Affiliations:** 1 Novo Nordisk Foundation Center for Protein Research, Faculty of Health and Medical Sciences, University of Copenhagen, Copenhagen, Denmark; 2 Section for Visual Computing, Department of Applied Mathematics and Computer Science, Technical University of Denmark, Lyngby, Denmark; 3 Department of Clinical Immunology, Faculty of Health and Medical Sciences, Rigshospitalet, University of Copenhagen, Copenhagen, Denmark; 4 Department of Cardiology, Faculty of Health and Medical Sciences, Rigshospitalet, The Heart Center, University of Copenhagen, Copenhagen, Denmark; 5 Department of Clinical Medicine, Faculty of Health and Medical Sciences, University of Copenhagen, Copenhagen, Denmark; 6 Department of Cardiology, Faculty of Health and Medical Sciences, University of Copenhagen, Herlev-Gentofte Hospital, Hellerup, Denmark; 7 Department of Cardiology, Gødstrup Hospital, Herning, Denmark; 8 Institute of Clinical Medicine, Aarhus University, Aarhus, Denmark

**Keywords:** Coronary angiography, Coronary artery disease, Ischemic heart disease, Deep learning, Quantitative coronary angiography, Myocardial infarction

## Abstract

The initial evaluation of stenosis during coronary angiography is typically performed by visual assessment. Visual assessment has limited accuracy compared to fractional flow reserve and quantitative coronary angiography, which are more time-consuming and costly. Applying deep learning might yield a faster and more accurate stenosis assessment. We developed a deep learning model to classify cine loops into left or right coronary artery (LCA/RCA) or “other”. Data were obtained by manual annotation. Using these classifications, cine loops before revascularization were identified and curated automatically. Separate deep learning models for LCA and RCA were developed to estimate stenosis using these identified cine loops. From a cohort of 19,414 patients and 332,582 cine loops, we identified cine loops for 13,480 patients for model development and 5056 for internal testing. External testing was conducted using automated identified cine loops from 608 patients. For identification of significant stenosis (visual assessment of diameter stenosis > 70%), our model obtained a receiver operator characteristic (ROC) area under the curve (ROC-AUC) of 0.903 (95% CI: 0.900–0.906) on the internal test. The performance was evaluated on the external test set against visual assessment, 3D quantitative coronary angiography, and fractional flow reserve (≤ 0.80), obtaining ROC AUC values of 0.833 (95% CI: 0.814–0.852), 0.798 (95% CI: 0.741–0.842), and 0.780 (95% CI: 0.743–0.817), respectively. The deep-learning-based stenosis estimation models showed promising results for predicting stenosis. Compared to previous work, our approach demonstrates performance increase, includes all 16 segments, does not exclude revascularized patients, is externally tested, and is simpler using fewer steps.

**Supplementary Information:**

The online version contains supplementary material available at 10.1007/s10554-025-03324-x.

## Introduction

X-ray multi-frame images (also known as cine loops, videos, views, or projections) acquired during an invasive coronary angiography (CAG) yield detailed information about the anatomy and flow in the coronary arteries [1]. Cine loops are acquired separately for the left coronary artery (LCA) and right coronary artery (RCA), and views are acquired from different angulations. During and after recordings, coronary angiographies (CAGs) are visually assessed to identify and quantify stenosis on all 16 coronary artery segments [2]. This visual assessment, often called "eyeballing", involves assessing the diameter reduction of the artery segment compared to the proximal reference in percentage. Based on the presence of stenoses, the need for pharmacotherapy and revascularization can be considered [2].

The visual assessment of a stenosis has a high observer variance [2, 3]. Recent guidelines suggest unnecessary use of percutaneous coronary intervention (PCI) and coronary artery bypass grafting (CABG) in 1–2% and 10–15% of cases, respectively, which is likely caused by inaccurate assessment of stenoses [2, 3].

Objective stenosis assessment can be evaluated by fractional flow reserve (FFR) measurements during the procedure, measuring the pressure drop across a stenosis to determine the hemodynamic significance of a stenosis. Despite the proven benefits of wire-based FFR measurements [4–9], utilization varies across hospitals and countries, with a utilization span between 5 and 17% [10–13]. The underutilization can be explained by the increased cost, time-requirement, patients discomfort, and additionally that FFR is not always technically possible [12]. Alternatively, quantitative coronary angiography (QCA) can be used for objective measurements of a vessel diameter reduction using image analysis software. QCA relies typically on manual keyframe extraction, manual segmentation of vessels with stenosis, followed by 3D reconstruction using two different angulations [14, 15]. This process is slow and requires specialized training. While FFR is considered the ground truth for determining hemodynamically significant stenosis, QCA is attractive for research as it can be performed offline and after the CAG [14]. It has been shown that revascularization of non-culprit lesions based on QCA can reduce future incidences of myocardial infarction [15]. Unfortunately, both QCA and FFR are expensive, time-consuming, and require special training to produce reliable results.

Considering these challenges in current stenosis assessment methods, there has been growing interest in applying deep-learning-based-approaches for automatic stenosis estimation [16–23]. Previous work, such as CathAI and DeepCoro, using reasonably sized datasets, has presented a complex pipeline with six steps and eight models, focusing only on 11 segments, excluding patients with prior revascularization [22, 23]. Crucial, the previously published work has not been externally tested and comprehensive evaluated against FFR or QCA. As a result, the task of stenosis estimation from coronary angiography remains unresolved.

In this paper, we present an end-to-end learning-based approach aiming to provide a useful clinical tool that could provide faster diagnoses and reduce costs. Our method has improved performance compared to related work, capable of estimating stenosis on all 16 segments without exclusion of patients with prior revascularization, and the performance was evaluated on an external test set from a different hospital. Furthermore, the performance was evaluated against both visual assessments, QCA, and FFR.

## Materials and methods

### Cohort description

#### Rigshospitalet dataset: Cohort description

Our dataset used for model development and testing included 19,414 patients, comprising 332,582 X-ray cine loops, extracted from Rigshospitalet, Copenhagen, (period 2006–2016). In total, the dataset contained 23,415 CAGs, and each CAG contained an average of 17.8 cine loops. The characteristics of the 19,414 patients, corresponding to the time point of coronary angiography, are presented in Table 1. CAGs were recorded using Philips Medical Systems, GE HealthCare, and Siemens Healthineers angio systems. The CAGs were linked to the Eastern Denmark Heart Registry (EDHR) database. The EDHR database contains information about visual assessment in each of the three major coronary arteries, reported according to the 16-segment classification protocol [24–26]. Additionally, the indication for coronary angiography and the treatment was recorded (Supplemental Table S1). Segments displaying borderline or intermediate stenosis were, if appropriate, further evaluated using Fractional Flow Reserve (FFR). Every entry in the EDHR database was manually registered by interventional cardiologists as part of clinical practice. We used 14,235 randomly selected patients for model development (approximately 90% for the training set with 12,846 patients and 10% for the validation set with 1389 patients). For evaluation of the model performance, we used 5179 randomly selected patients for evaluating the performance, which we will refer to as the internal test set (see also Supplemental Materials Fig. S2 for addtional information).

**Table 1:** Cohort characteristics

Features	Total
Patients	19,414
Age, years	67.3 ± 12.4
Males (%)	13,377 (68.9%)
Diabetes (%)	3634 (18.7%)
Hypertension (%)	10,042 (51.7%)
Smokers and ex-smokers (%)	12,752 (65.7%)
No vessel abnormalities (%)	2892 (14.9%)
Atheromatous vessels (%)	4886 (25.2%)
1 vessel disease (%)	7030 (36.2%)
2 vessel disease (%)	3477 (17.9%)
3 vessel disease (%)	3209 (16.5%)
Left main disease (%)	1094 (5.6%)
Prior PCI (%)	4124 (21.2%)
Prior CABG (%)	1506 (7.7%)
Right dominant (%)	15,539 (80.0%)
Left dominant (%)	1748 (9.0%)
Co dominant (%)	1886 (9.7%)
Missing information about dominance (%)	241 (1.3%)
Arrhythmia device (%)	827 (4.3%)

*PCI*  percutaneous coronary intervention, *CABG*  coronary artery bypass grafting

#### Skejby Hospital: Cohort for external testing

We further evaluated the model on 608 patients from Skejby Hospital in the Central Denmark Region, which we refer to as the external test set. These patients were selected following initial findings of suspicious stenosis from coronary computed tomography angiography (CTA). Each patient had a single coronary angiography recorded using Philips Medical Systems and Siemens Healthineers Angio System scanners. FFR was measured in all segments technically feasible for FFR measurements. All applicable segments were also analyzed with QCA (qantitative coronary angiography) using Medis QAngio®XA 3D, Netherlands. No additional patient characteristics such as demography were available for the external test dataset.

### Overview: a deep learning-based approach for automated stenosis estimation

**Fig. 1:**
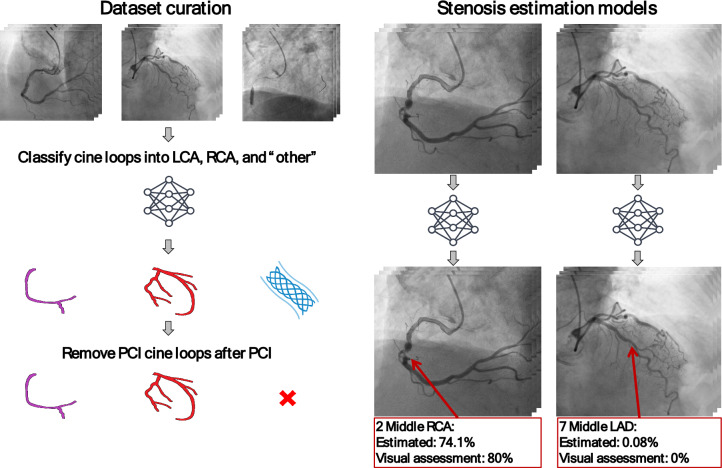
Overview of the approach developed for stenosis estimation

We employed a multi-stage approach for estimating the degree of stenosis on all coronary artery segments. First, we manually annotated a subset of cine loops as either left or right coronary arteries (LCA/RCA). Secondly, we developed and trained a deep learning model to differentiate between LCA and RCA in coronary angiography cine loops. This model was used to classify all cine loops as LCA, RCA, or "other". Thirdly, we selected all cine loops before revascularization using an automated approach based on the classified cine loops and the timestamp. Fourthly, we developed two deep learning models for estimating stenosis: one for LCA and another for RCA, utilizing the cine loops before revascularization. An overview of the approach can be found in Fig. 1.

### R2D + 1 backbone deep learning model

We used the R2D + 1 deep learning model [27], a supervised 3D convolutional neural network (CNN), which has previously demonstrated state-of-the-art performance for CAG cine loops classification of right and left coronary artery [28, 29]. The R2D + 1 model uses the R2D + 1 block, which compresses the 3D convolutional block into a spatial block with filters of size 3 × 3 × 1 and a temporal block with filters of size 1 × 1 × 3. Non-linear ReLu activation is used between the spatial and temporal filters. The R2D + 1 block can be interpreted as a combination of spatial and temporal filters, but with non-linearity between the two operations, extracting non-linear relations between spatial and temporal features. The R2D + 1 network consists of R2D + 1 blocks and pooling, forming a hierarchical architecture designed to learn spatial and temporal features across different scales.

For both CAG cine loop classification tasks and stenosis estimation, we employed the R2D + 1 network. The model takes a CAG cine loop as input from the training set and learns discriminative features. The discriminatory features will depend on the target used for the model, and thus, the model learns different features for the cine loop classification model and stenosis estimation models.

### Annotation of cine loops

To categorize all the cine loops, we manually labelled a subset of 18,058 cine loops from 1228 patients as LCA, RCA and "other". The "other" category included cine loops, in which the LCA or the RCA was not present. The purpose of the "other" category was to exclude cine loops not relevant for visual assessment. We specifically categorized cine loops containing guide wires as "other", even when they also displayed either the left or the right coronary artery. Cine loops containing chronic total occlusions (CTO) were still annotated as LCA or RCA. For training and validation, we used 1047 patients with 15,068 cine loops. For model evaluation, we used a test dataset of 2990 cine loops from 179 patients (Supplemental Table S3).

### Cine loop classification model

We developed a deep learning classification model designed to classify cine loops into one of the three categories: LCA, RCA and "other" using the labeled subset. We used the trained cine loop classification model to categorize the cine loops in the training/validation and the test sets as LCA (LAD and LCX), RCA and "other". This classification step extends the work of Eschen et al. [28, 29], who focused on cine loop classification of left and right coronary arteries, by incorporating an additional "other" category. This extension provides a more comprehensive categorization of cine loops, enhancing its utility for cine loop curation by filtering out cine loops that are not relevant for stenosis assessment.

### Diagnostic cine loop selection

The cine loops obtained during and post revascularization are not applicable to the decision-making process regarding revascularization in a deployment scenario of the models. Additionally, cine loops obtained during and post revascularization are highly associated with stenoses and may, therefore, introduce bias in the model during training. Consequently, we excluded cine loops performed during and post revascularization procedures. This exclusion involved removing cine loops categorized as "other" and any cine loops obtained after this category appeared in the sequence based on their timestamps. We denote this step as the "diagnostic cine loop selection step" as depicted in the Fig. 1. A detailed explanation of the data inclusion process is presented in Supplemental Materials Sect. 1.1, and Fig. S1.

### Training the stenosis estimation models

**Fig. 2:**
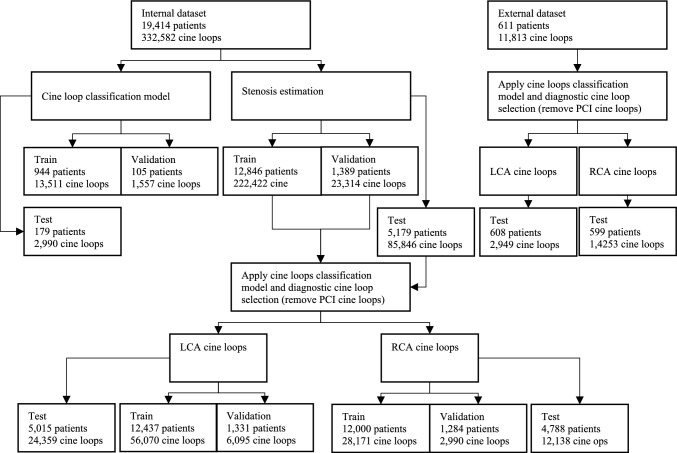
Overview of the different data partitions used for model development and testing. The curated dataset for model development included 13,840 patients, which was the union of the LCA and the RCA cine loops for training and validation. The curated internal test set included cine loops from 5056 patients (the union of LCA and RCA cine loops). Similarly, the curated external test set contained cine loops from 608 patients

Using the diagnostic cine loop selection procedure, we included cine loops of LCA and RCA and excluded cine loops obtained during and after PCI. The selection procedure resulted in 13,284 patients with 31,161 RCA cine loops, and 13,768 patients with 62,165 LCA cine loops (see Fig. 2, Supplemental Materials Table S4, and Fig. S2–S3 for details). We developed the stenosis estimation models individually for RCA and LCA using these 31,161 and 62,165 cine loops.

For both models, we used multi-output regression models. For the RCA stenosis estimation model, the final linear layer contained five neurons, one for each of the five RCA segments. Specifically, for the RCA model, the five output neurons corresponded to artery segments relevant to the RCA. Similarly, for the LCA stenosis estimation model, we used a multi-output regression model with 13 neurons in the final linear layer, one for each of the 13 segments relevant to the LCA (we also include the Posterior Descending Artery (PDA) and the Posterior Left Ventricular Artery (PLA) in the LCA model). This design ensures that the model can simultaneously make stenosis estimates for each segment, making it capable of handling multiple stenoses at once.

As the visual stenosis assessment was only reported for segments with identified stenosis, we interpreted the missing values as zeros, corresponding to segments without stenosis. Therefore, we had a complete dataset that included cine loops and corresponding visual assessment of stenosis on all coronary artery segments.

### Evaluating stenosis estimation models against visual assessment

Using the diagnostic cine loop selection procedure, we established an internal test set with 5056 patients (24,359 cine loops of the LCA from 5015 patients and 12,138 cine loops of the RCA from 4788 patients, as shown in Fig. 2, and Supplemental Fig. S4). Additionally, we leveraged the external cohort with 608 patients for external test (2949 cine loops of LCA from 608 patients and 1425 cine loops of RCA from 599 patients as depicted in Supplemental Fig. S5).

The final LCA and RCA stenosis estimates were obtained by selecting the most severe stenosis estimate (the maximum stenosis) from all cine loops in a CAG examination. Coronary dominance was used to decide whether LCA or RCA predictions should be employed to evaluate the PDA and PLA segments.

We evaluated the model’s ability to predict diameter stenosis as a continuous outcome. We also assessed its ability to distinguish between significant and non-significant stenosis as a binary outcome. We applied the clinical threshold for significant coronary artery diameter stenosis > 70%, except for the left main segment, which was > 50% [25]. We assessed the performance of the stenosis predictions for each of the 16 segments of the LCA and RCA models, as well as the overall average performance.

We also evaluated the stenosis estimation model using our “Angina-FFR Subset”. This subset consisted of 499 patients with similar clinical characteristics compared to those in the external test set, also including patients with FFR measurements in at least one segment, atheromatous lesions, and single-vessel or two-vessel disease.

### Evaluating stenosis estimation models against FFR

For the subset of angiographies followed by FFR measurements (1180 patients in the internal test set and 439 patients in the external test set), we compared the stenosis estimates against FFR measurements. FFR values, our stenosis estimates, visual assessments, and QCA appear on a scale from 0 to 1. For FFR, values close to 1 indicates no ischemia. In contrast, higher values (i.e., close to 1) from our estimated stenosis, visual assessments, and QCA indicate increased stenosis diameter, which is associated with ischemia. Therefore, the scales are inversely related. To align these scales, we transformed the stenosis estimates, visual assessments, and QCA values by subtracting them from 1, ensuring comparability with the FFR measurements. Using this transformed scale, we compared these values against FFR as a continuous variable. We also evaluated the performance on detecting hemodynamic significant stenosis (FFR ≤ 0.8). To establish a comparable baseline for predicting FFR ≤ 0.8, we evaluated the performance using visual assessments as predictors.

### Evaluating stenosis estimation models against QCA

The estimated stenosis was also compared against QCA in the external test set for 359 patients. The evaluation was performed similarly to the evaluation against visual assessment. As we had access to both the visual assessments and FFR in this dataset for 209 of the patients, we established a baseline for comparison using visual assessment and FFR as predictors for QCA.

### Statistical analysis

The estimated stenosis was compared against visual assessment, FFR, and QCA measurements using mean absolute error (MAE) and the Pearson's correlation coefficient (r). The estimated stenoses were also compared against FFR using these metrics.

To evaluate the performance on detecting significant stenoses, we used the area under the Receiver Operating Characteristic curve (ROC AUC), the area under the precision-recall curve (PR AUC), F1 score, precision, sensitivity, and specificity. The confidence intervals were computed using 1000 bootstrap samples at a 95% confidence level defined by the 2.5th and 97.5th percentiles. To determine whether two predictors (e.g., estimated stenosis versus visual assessment in predicting hemodynamically significant stenosis measured by FFR) were statistically different, confidence intervals of the differences were computed using 1000 bootstrap samples. If the resulting confidence interval contained zero, the difference between the predictors was not considered statistically significant.

## Results

The cohort characteristics for the internal dataset are presented in Table 1, and a flowchart describing the flow of cine loops and patients for the different data subsets is presented in Fig. 2.

### Performance of the cine loop classification model

The performance of the cine loop classification model had a macro F1 score of 0.972 (95% CI: 0.972–0.972) on the internal test set). We assessed the discordant predictions (79 cine loops identified as the off-diagonal of the confusion matrix in Supplemental Materials Fig. S6) and found that most of these originated from cases with ambiguous labels (e.g., cine loops obtained while measuring the FFR using a guide wire).

### Performance of the stenosis estimation model

For predicting the visual assessment (diameter stenosis), we obtained a MAE of 0.178 (95% CI 0.177–0.179), and a Pearson's correlation coefficient of 0.661 (95% CI 0.656–0.666) on the internal test set. On the “Angina-FFR Subset" we obtained an MAE of 0.156 (95% CI: 0.144–0.168), and Pearsons's correlation coefficient of 0.293 (95% CI: 0.196–0.393) when predicting visual assessment of stenosis. On the external test set, we obtained an MAE of 0.186 (95% CI: 0.182–0.190) and a Pearson's correlation coefficient of 0.386 (95% CI: 0.317–0.373) compared to the visual assessment.

**Fig. 3:**
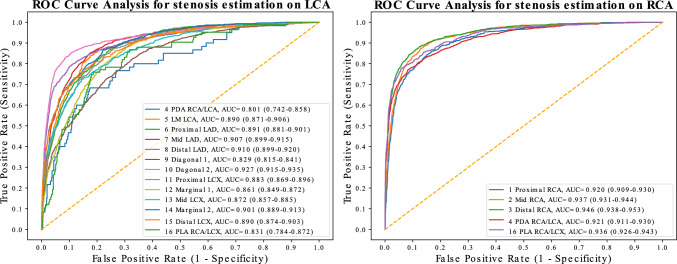
ROC curve for significant stenosis detection for each segment on the internal test set (visual assessment of diameter stenosis > 70%)

We evaluated the model's performance on significant stenosis identification and obtained a ROC AUC of 0.903 (95% CI: 0.900–0.906), and PR AUC of 0.693 (95% CI: 0.685–0.701), as seen in Fig. 3. On the "Angina-FFR Subset" we obtained a ROC AUC of 0.849 (95% CI: 0.829–0.867), PR AUC of 0.486 (95% CI: 0.436–0.530) when predicting significant stenoses.

**Fig. 4:**
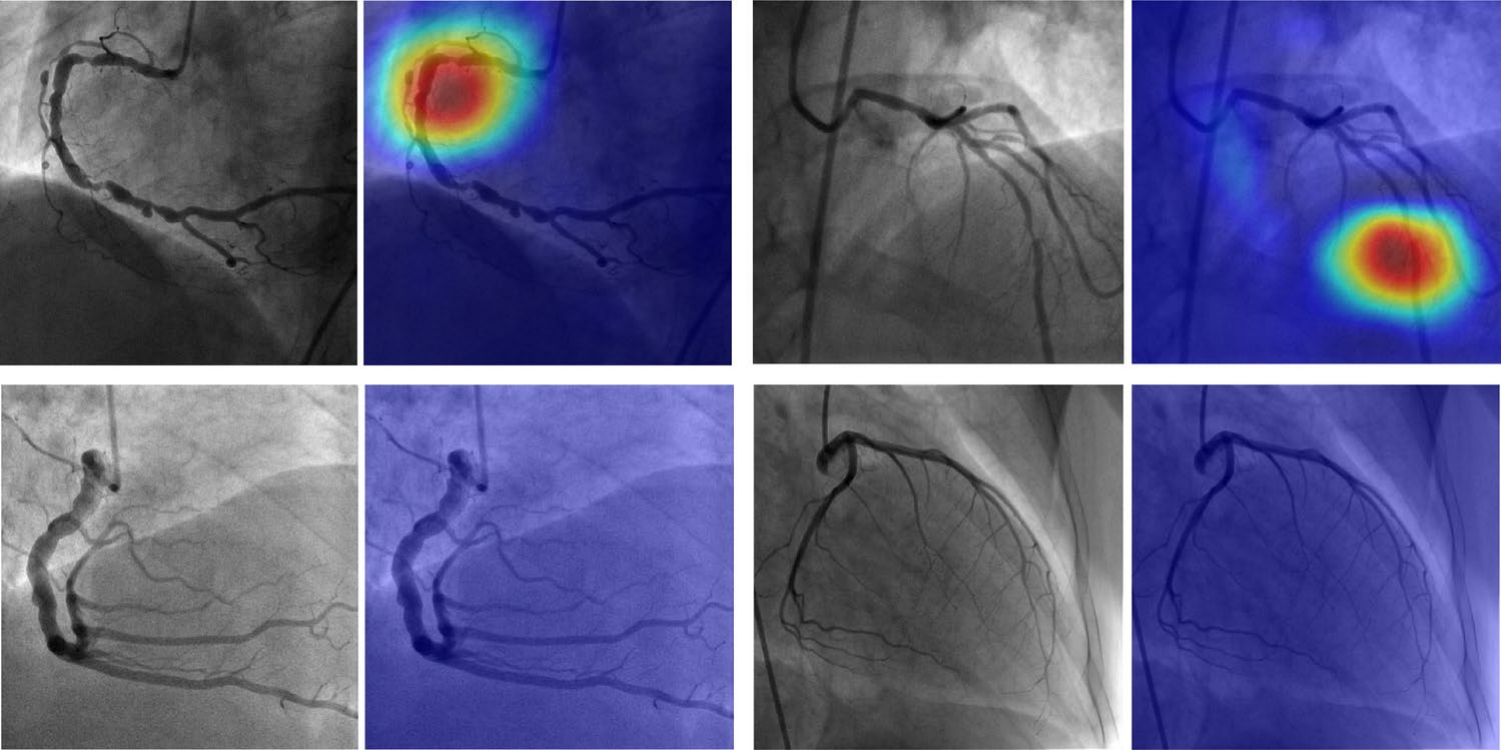
Saliency maps highlighting regions influencing the predictions obtained using Gradient-based Class Activation Maps (Grad-CAM) [31]. The top left image highlights the saliency regions in the cine loop for predicting stenosis in the proximal RCA, where the model predicted 81.6% stenosis, and the cardiologist reported 70%. In the top right image, the model predicted 61.0% stenosis, while the cardiologist reported 80% stenosis in the middle LAD segment. Stenoses were also present in the middle RCA, distal RCA, and left main, however, their corresponding saliency maps are not shown. The two bottom images represent cases with no stenosis

For detection of significant stenosis on the external test set, the ROC AUC decreased to 0.833 (95% CI: 0.814–0.852), and PR AUC decreased to 0.219 (95% CI: 0.190–0.250) as shown in Table 2 (the performances on the individual segments are depicted in Supplemental Materials Tables S5–S6). Figure 4 presents saliency maps highlighting regions influencing the stenoses predictions. Additional performance metrics using MemVIT architecture [30] instead of the R2D + 1 are presented in Supplemental Materials Table S13.

**Table 2:** Performance on predicting visual assessment

	Internal test set	External test set
Method	Estimated stenosis (this paper)	Estimated stenosis (this paper)
MAE	0.178 (0.177–0.179)	0.186 (0.182–0.190)
*r*	0.661 (0.656–0.666)	0.345 (0.317–0.373)
ROC AUC	0.903 (0.900–0.906)	0.833 (0.814–0.852)
PR AUC	0.693 (0.685–0.701)	0.219 (0.190–0.250)
F1	0.637 (0.631–0.643)	0.314 (0.284–0.343)
Sensitivity	0.681 (0.674–0.689)	0.548 (0.503–0.593)
Specificity	0.922 (0.920–0.924)	0.901 (0.895–0.907)
Precision	0.599 (0.591–0.606)	0.220 (0.199–0.245)

The F1 score, sensitivity, specificity, and precision were obtained using the threshold of 0.361 on the estimated stenosis. The threshold of 0.361 was chosen to maximize the F1 score on predicting visual assessment of stenosis > 70% using the validation set

*MAE* mean absolute error, *r* Pearsons correlations coefficient, *ROC AUC* receiver operator characteristics area under the curve, *PR AUC* Precision-recall area under the curve

### Predicting fractional flow reserve

For predicting the measured FFR values on the internal test set, we obtained an MAE of 0.157 (95% CI 0.148–0.165) and a Pearson's correlation coefficient of 0.220 (95%CI 0.163–0.281). On the external test set, we obtained an MAE of 0.120 (0.111–0.129) and a Pearson's correlation coefficient of 0.441 (95% CI 0.375–0.502) when predicting the measured FFR values.

For the detection of hemodynamically significant stenosis (FFR ≤ 0.80), we obtained a ROC AUC of 0.651 (95% CI: 0.616–0.686) on the internal test set, and a ROC AUC of 0.780 (95% CI: 0.743–0.817) on the external test set as shown in Table 3 (performance on individual segments are depicted in Tables S7–S10 in Supplemental Materials).

**Table 3:** Performance on predicting FFR

	Internal test set				External test set			
Method	Estimated stenosis (this paper)	Visual assessment	Estimated stenosis vs visual assessment	Advantage	Estimated stenosis (this paper)	Visual assessment	Estimated stenosis vs visual assessment	Advantage
MAE	0.157 (0.148 to 0.165)	0.394 (0.385 to 0.401)	(− 0.244 to − 0.223)	Estimated stenosis	0.120 (0.111 to 0.129)	0.254 (0.240 to 0.268)	(− 0.145 to − 0.117)	Estimated stenosis
r	0.220 (0.163 to 0.281)	0.549 (0.494 to 0.598)	(− 0.399 to − 0.260)	Visual assessment	0.441 (0.375 to 0.502)	0.640 (0.600 to 0.676)	(− 0.275 to − 0.139	Visual assessment
ROC AUC	0.651 (0.616 to 0.686)	0.853 (0.828 to 0.876)	(− 0.267 to − 0.187)	Visual assessment	0.780 (0.743 to 0.817)	0.848 (0.817 to 0.877)	(− 0.124 to − 0.040)	Visual assessment
PR AUC	0.400 (0.353 to − 0.452)	0.635 (0.583 to 0.685)	(− 0.326 to − 0.212)	Visual assessment	0.441 (0.365 to 0.524)	0.532 (0.449 to 0.606)	(− 0.208 to − 0.052)	Visual assessment
F1	0.417 (0.368 to 0.465)	0.680 (0.636–0.723)	(− 0.216 to 0.075)	No difference	0.438 (0.361 to 0.511)	0.558 (0.497 to 0.621)	(− 0.109 to 0.072)	No difference
Sensitivity	0.411 (0.360 to 0.465)	0.690 (0.638 to 0.742)	(− 0.103 to 0.046)	No difference	0.386 (0.311 to 0.464)	0.611 (0.526 to 0.689)	(− 0.040 to 0.132)	No difference
Specificity	0.786 (0.761 to 0.814)	0.869 (0.847 to 0.892)	(− 0.220 to − 0.160)	Visual assessment	0.914 (0.891 to 0.936)	0.868 (0.842 to 0.894)	(− 0.082 to − 0.031)	Visual assessment
Precision	0.427 (0.372 to 0.480)	0.670 (0.619 to 0.716))	(− 0.476 to − 0.339)	Visual assessment	0.510 (0.423 to 0.594)	0.519 (0.445 to 0.590)	(− 0.306 to − 0.083)	Visual assessment

To compare the estimated stenosis, the visual assessments, and the QCA values against FFR values, these values were transformed to a comparable scale by subtracting the values from 1. The F1 score, sensitivity, specificity, and precision were obtained using the threshold of 0.361 on the estimated stenosis. The threshold of 0.361 was chosen to maximize the F1 score on predicting visual assessment of stenosis > 70% using the validation set

*MAE* mean absolute error, *r* Pearsons correlations coefficient, *ROC AUC* receiver operator characteristics area under the curve, *PR AUC* Precision-recall area under the curve, *QCA* quantitative coronary angiography

### Predicting QCA

We further evaluated the performance on QCA prediction on the external test set (QCA was not measured in the dataset from Rigshospitalet). We obtained a MAE of 0.210 (95% CI 0.203–0.217) and a Pearson's correlation coefficient of 0.477 (95% CI 0.423–0.530). For detection QCA diameter stenosis > 70%, we obtained a ROC AUC of 0.798 (95% CI: 0.782–0.814), as depicted in Table 4. For detecting QCA-based significant stenosis, our models were consistently better than visual assessment and FFR with a ROC AUC of 0.798 versus 0.658 and 0.575 (additional performance metrics on individual segments are depicted in Tables S11–S12 in Supplemental Materials).

**Table 4:** Performance for predicting QCA on the external test set

Method	Estimated stenosis (this paper)	Visual assessment	Estimated stenosis vs visual assessment	Advantage	FFR	Estimated stenosis vs FFR	Advantage
MAE	0.210 (0.203 to 0.217)	0.351 (0.343 to 0.360)	(− 0.148 to − 0.130)	Estimated stenosis	0.265 (0.248 to 0.285)	(− 0.145 to − 0.093)	Estimated stenosis
r	0.477 (0.423–0.530)	0.358 (0.302 to 0.408)	(0.050 to 0.17)	Estimated stenosis	0.180 (0.037 to 0.325)	(− 0.015 to 0.21)	No difference
ROC AUC	0.798 (0.741 to 0.842)	0.658 (0.591 to 0.726)	(0.087 to 0.218)	Estimated stenosis	0.575 (0.491 to 0.663)	(− 0.021 to 0.207)	No difference
PR AUC	0.340 (0.243 to 0.443)	0.273 (0.179 to 0.374)	(− 0.029 to 0.202)	No difference	0.430 (0.318 to 0.552)	(− 0.088 to 0.155)	No difference
F1	0.246 (0.188 to 0.304)	0.246 (0.183 to 0.312)	(− 0.11 to 0.026)	No difference	0.058 (0.000 to 0.143)	(0.228 to 0.482)	Estimated stenosis
Sensitivity	0.578 (0.471 to 0.688)	0.446 (0.340 to 0.563)	(0.022 to 0.296)	Estimated stenosis	0.030 (0.000 to 0.077)	(0.206 to 0.429)	Estimated stenosis
Specificity	0.817 (0.797 to 0.836)	0.872 (0.853 to 0.889)	(− 0.115 to − 0.074)	Visual assessment	0.985 (0.962 to 1.000)	(− 0.213 to 0.087)	No difference
Precision	0.157 (0.116 to 0.198)	0.170 (0.123 to 0.225)	(− 0.119 to 0.013)	No difference	0.488 (0.000 to 1.000)	(− 0.522 to 0.575)	No difference

*MAE* mean absolute error, *r* Pearsons correlations coefficient, *ROC* AUC receiver operator curve area under the curve, *PR AUC* Precision-recall area under the curve, *FFR* fractional flow reserve

The F1 score, sensitivity, specificity, and precision were obtained using the threshold of 0.361 on the estimated stenosis. The threshold of 0.361 was chosen to maximize the F1 score on predicting visual assessment of stenosis > 70% using the validation set

## Discussion

Our deep learning model demonstrated robust performance in classifying cine loops into LCA, RCA, and "other" categories, with a macro F1 score of 0.972. For detecting significant stenosis, high ROC AUC levels of 0.903 on the internal test set and of 0.833 on the external test set were found. The model outperformed visual assessment when validated against QCA, achieving a ROC AUC of 0.798. For predicting hemodynamically significant stenosis measured by FFR, the model achieved a ROC AUC of 0.651 on the internal test set and 0.780 on the external test set. Here, we discuss our findings regarding the related works, visual assessments, FFR, and QCA, and finally, we address the limitations of our approach.

### Related works

In recent years, several studies have focused on the significant stenosis detection in coronary angiography (CAG) cine loops. Although many of these advancements have been based on small datasets only considering single CAG frames [16–18], efforts for significant stenosis detection on larger datasets exist [19–23]. For instance, Avram et al. curated and trained a model (CathAI) including 11,972 patients, achieving a ROC AUC of 0.839 on an internal test set [19]. Most recently, and comparable to our work, Langlais et al. introduced DeepCoro, developed by the same research group behind CathAI. DeepCoro is a 6-step pipeline that includes primary structure identification, stenosis detection, frame registration, coronary artery segmentation, alignment of stenosis with segments, and finally, stenosis regression [23]. DeepCoro was developed using 182,418 coronary angiography cine loops, and it obtained a ROC AUC of 0.829 on stenosis detection, and a MAE of 20.15% on predicting visual assessment in percentage on an internal test set.

Our approach outperformed DeepCoro with a ROC AUC of 0.903 (0.900–0.906) and a PR AUC of 0.693 (0.685–0.685), compared to DeepCoro’s ROC AUC of 0.8294 (0.8215–0.8373) and DeepCoro’s PR AUC of 0.5239 (0.5041–0.5421). Notably, our approach involves 2 steps and 3 models, while DeepCoro uses 6 steps and 8 models [23].

Additionally, DeepCoro only focused on 11 segments, instead of 16 segments and excluded patients with prior CABG and PCI [23]. Both aspects are highly relevant for assessing a CAG [2]. Moreover, previous work did not evaluate the performance against FFR, and the performance was not evaluated on an external test set from another hospital. Finally, our methods can run on the fly with a processing speed of 0.03 s for a cine loop which is significantly better than DeepCoro with a processing speed of 62.6 s.

Hence, we aimed to address the limitations in existing work, our models obtain superior performance, and the approach uses a simpler and faster pipeline.

### Comparison against visual assessment

While we obtained the best performance reported in the literature for significant stenosis detection (ROC AUC of 0.903 and PR AUC of 0.693), we observed a notable performance drop on the external test set (ROC AUC of 0.833 and PR AUC of 0.219). A similar pattern emerged when evaluating the model on the "Angina-FFR Subset," where the ROC AUC decreased to 0.849 and the PR AUC to 0.486. The most significant factor contributing to this performance decline appears to be the difference in patient characteristics. The external test set consisted of patients selected based on prescreening with CTA, leading to a higher proportion of individuals with intermediate stenosis and excluding those with mild stenosis or multivessel disease, such as patients with STEMI or NSTEMI.

### Comparison against FFR

For predicting hemodynamically significant stenoses, our model achieved a ROC AUC of 0.651 (95% CI: 0.616–0.686) on the internal test set and 0.780 (95% CI: 0.743–0.817) on the external test set. The performance of the model was inferior on predicting hemodynamical significant stenosis compared to visual assessment, which achieved a ROC AUC of 0.853 and 0.844 on the test set and the external test set, respectively. As shown in Table 3, the estimated stenosis outperformed visual assessment only in terms of mean absolute error (MAE), with no significant differences in sensitivity or F1 score. Visual assessment performed better across the remaining metrics. However, the reported visual assessments can be overoptimistic and biased towards FFR measurements, as they are typically reported after the FFR is measured and are not blinded to it. While our models demonstrated good performance on predicting visual assessment, there is still room for improvement for predicting FFR, and hemodynamically significant stenosis, as indicated by the broader set of metrics reported in Table 3.

### Comparison against QCA

For detecting the clinically important threshold of QCA diameter stenosis > 70% on the external test set, our model achieved a ROC AUC of 0.798 (95% CI: 0.782–0.814). This performance was consistently better than visual assessment, which obtained a ROC AUC of 0.658 (95% CI: 0.591–0.726), and FFR, which obtained a ROC AUC of 0.575 (95% CI: 0.491–0.552). While there is strong evidence that FFR is optimal for revascularization decisions, QCA is attractive for research. As seen in Table 4, our model obtained better metrics compared to visual assessment when predicting QCA. Our methodology has the potential to be used as a fast and accurate alternative to traditional QCA. Another notable finding is that using FFR to determine significant stenosis from QCA yielded low performance.

### Limitations

Despite the promising results, we acknowledge several limitations in our results. The most notable limitation is that the stenosis estimation models were trained on patients undergoing routine coronary angiography, including patients without disease and those with multivessel disease. As a result, the stenosis estimation models are not guaranteed to generalize to patient cohorts with other inclusion/exclusion criteria (e.g., patients undergoing CTA before coronary angiography), which can be seen in the decreased performance on predicting visual assessment in the external test set and the subset "Angina-FFR Subset". Secondly, the stenosis estimation models were trained using visual assessments, as we did not have access to QCA, and FFR measurements being only available for borderline stenosis segments and where it was technically feasible to perform the measurements. Training on visual assessment from multiple observers or training on QCA or FFR targets would potentially improve performance.

## Conclusion

Our approach for stenosis estimation showed promising results, outperforming previous work on predicting visual assessments. However, a significant performance drop was observed in the external test cohort, which had suspected stenosis detected by CTA. Predicting hemodynamically significant stenosis measured by FFR using the stenosis estimation models did not surpass using visual assessments as predictors, indicating that improvements in this area are likely needed. Notably, the stenosis estimations were better at predicting QCA diameter stenosis compared to visual assessments. These results suggest that our approach for stenosis estimation is clinically relevant, offering a faster and more objective alternative to traditional methods. Future research should focus on improving the models and investigating the effect of the estimated values on the treatment compared with traditional methods.

## Data availability statement

Data access applications can be made to the Danish Health Data Authority (contact: servicedesk@sundhedsdata.dk). Anyone wanting access to the data and to use them for research will be required to meet research credentialing requirements as outlined at the authority's web site: https://sundhedsdatastyrelsen.dk/da/english/health_data_and_registers/research_services. Requests are normally processed within 3–6 months.

## Supplementary Information

Below is the link to the electronic supplementary material.

Supplementary file1 (PDF 48 KB)

Supplementary file2 (PDF 38 KB)

Supplementary file3 (PDF 37 KB)

Supplementary file4 (PDF 167 KB)

Supplementary file5 (PDF 78 KB)

Supplementary file6 (PDF 78 KB)

Supplementary file7 (PDF 78 KB)

Supplementary file8 (PDF 15 KB)

Supplementary file9 (PDF 186 KB)

Supplementary file10 (PDF 179 KB)

Supplementary file11 (PDF 56 KB)

Supplementary file12 (PDF 227 KB)

Supplementary file13 (DOCX 21333 KB)

Supplementary file14 (MP4 3993 KB)

Supplementary file15 (MP4 3338 KB)
