# Supplementary figures and images for: Automated stenosis estimation of coronary angiographies using end-to-end learning
Source: Int J Cardiovasc Imaging. 2025 Jan 9;41(3):441–52. doi: 10.1007/s10554-025-03324-x (PMC11880145; doi:10.1007/s10554-025-03324-x)

### ROC Curve Analysis on LCA

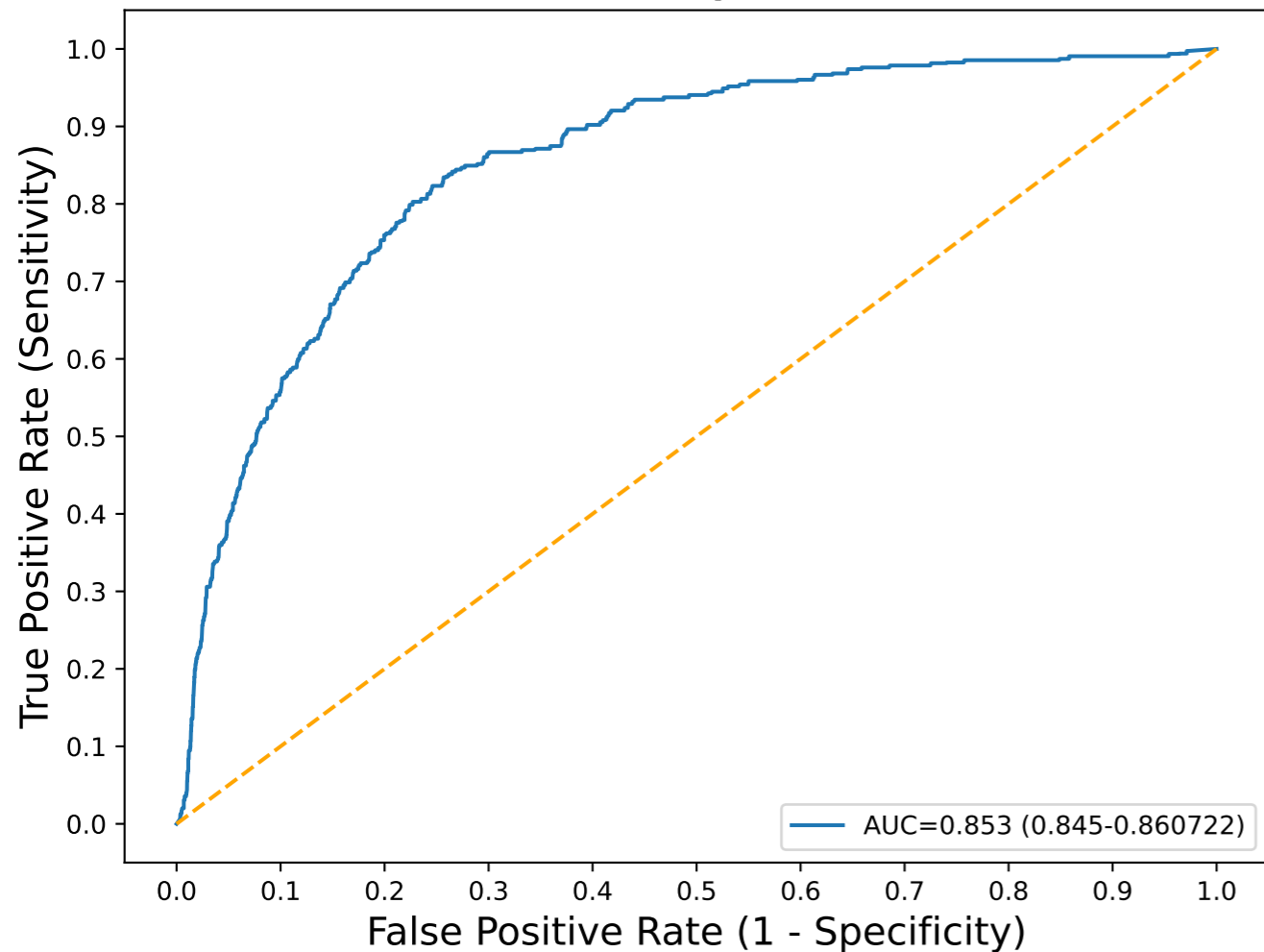

### ROC Curve Analysis on RCA

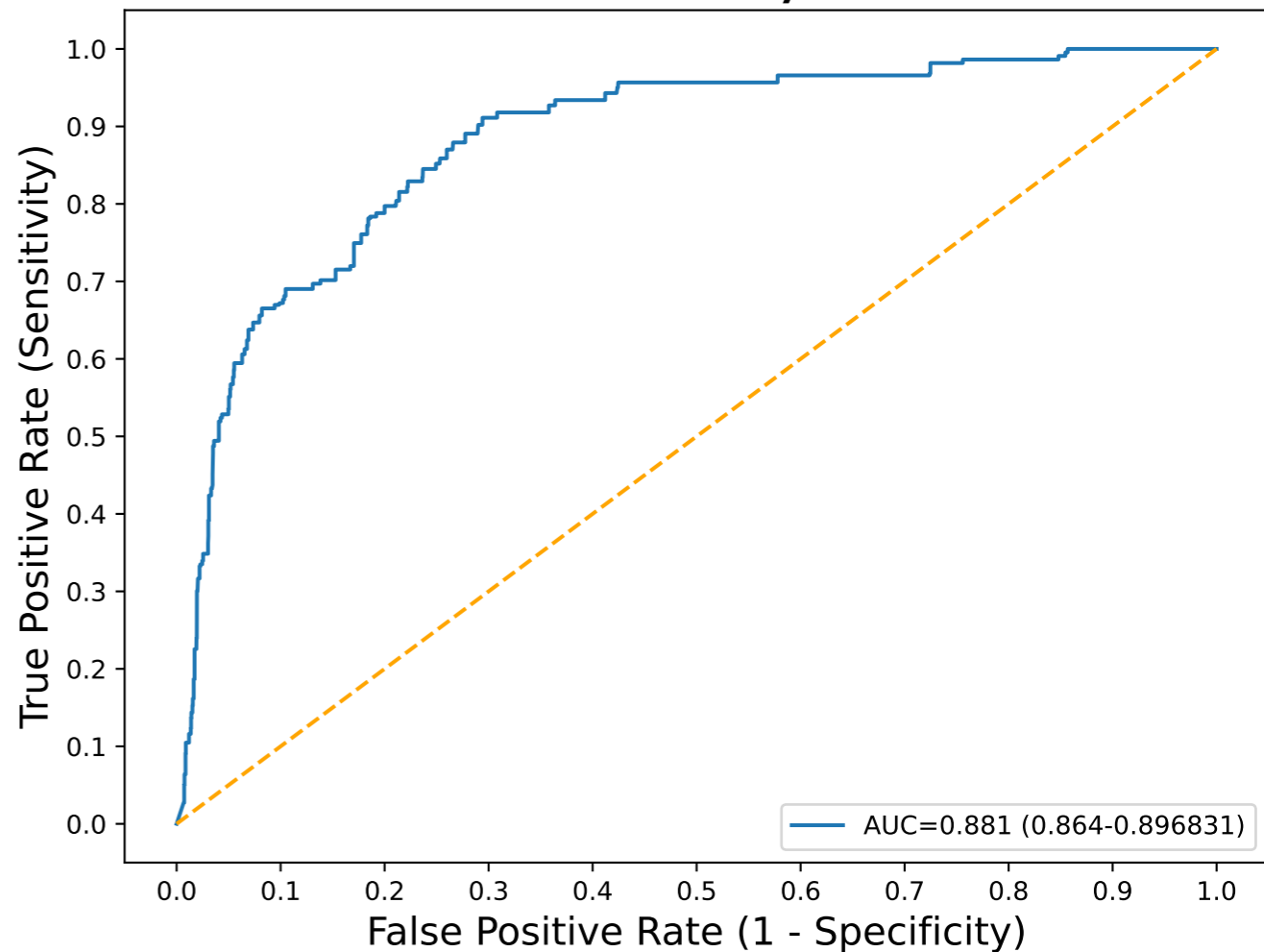

Supplement: Supplementary file 1 — Supplementary file1 (PDF 48 KB) [file 10554_2025_3324_MOESM1_ESM.pdf]

### ROC Curve Analysis on LCA

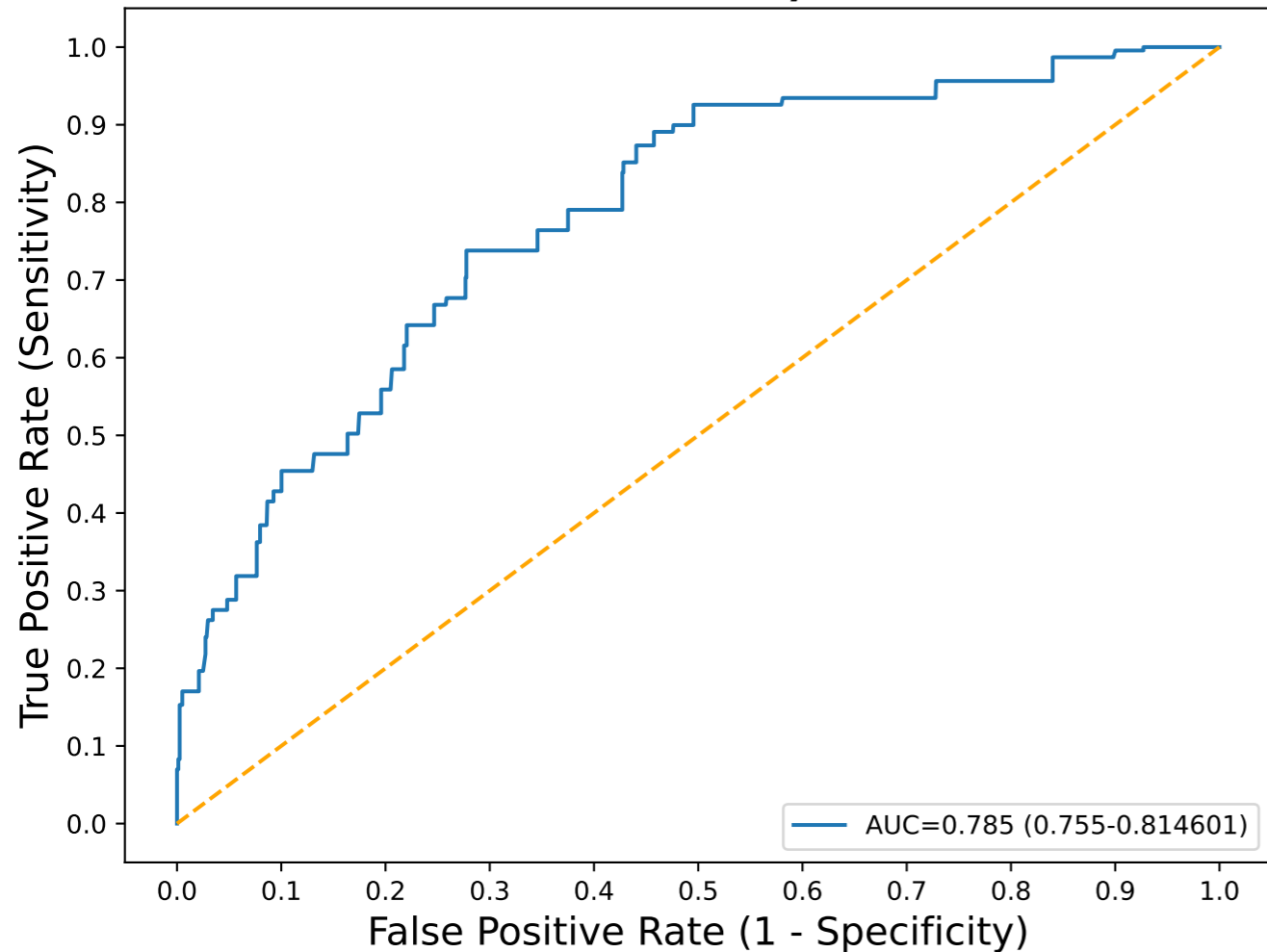

### ROC Curve Analysis on RCA

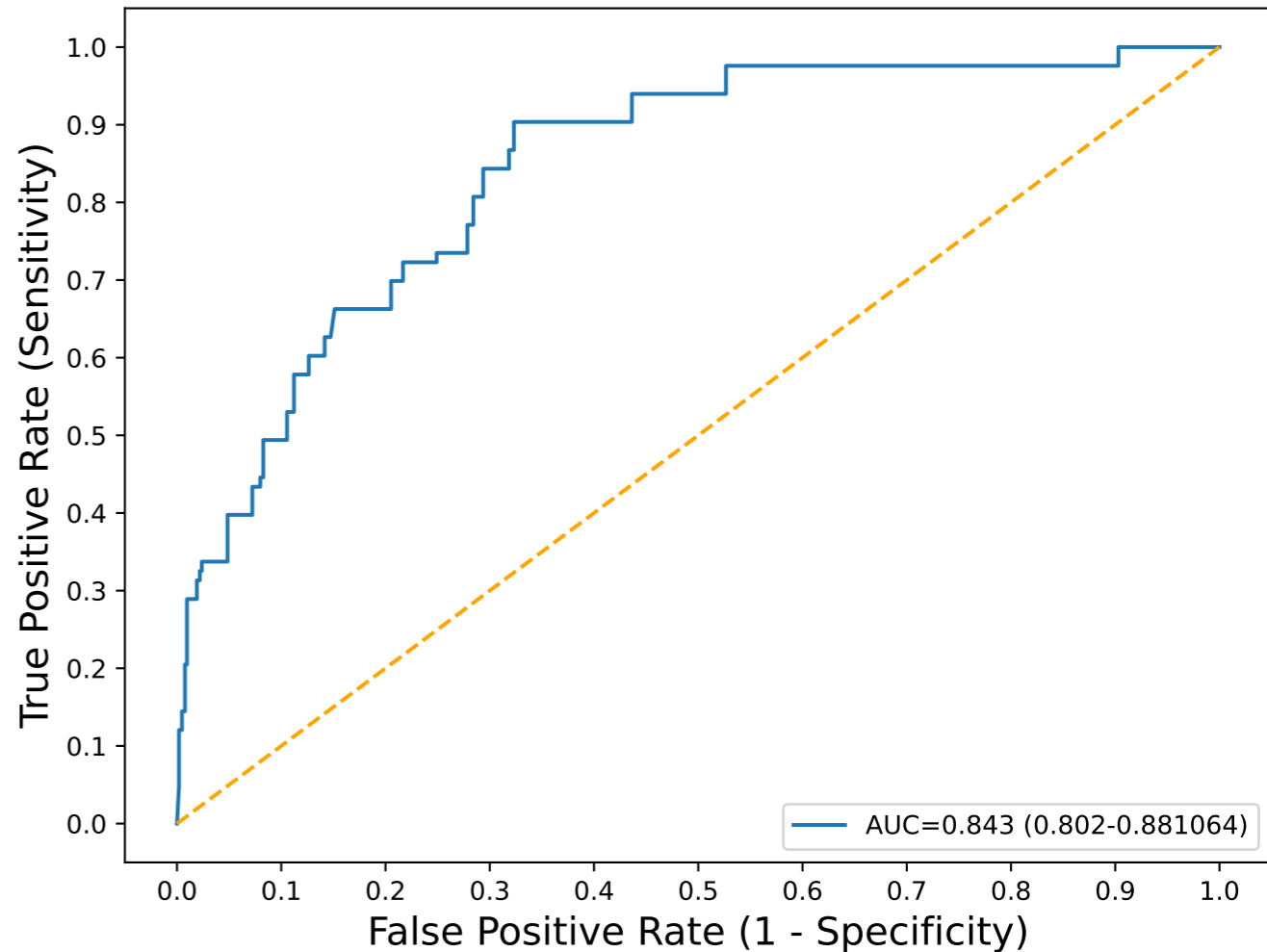

Supplement: Supplementary file 2 — Supplementary file2 (PDF 38 KB) [file 10554_2025_3324_MOESM2_ESM.pdf]

### ROC Curve Analysis LCA

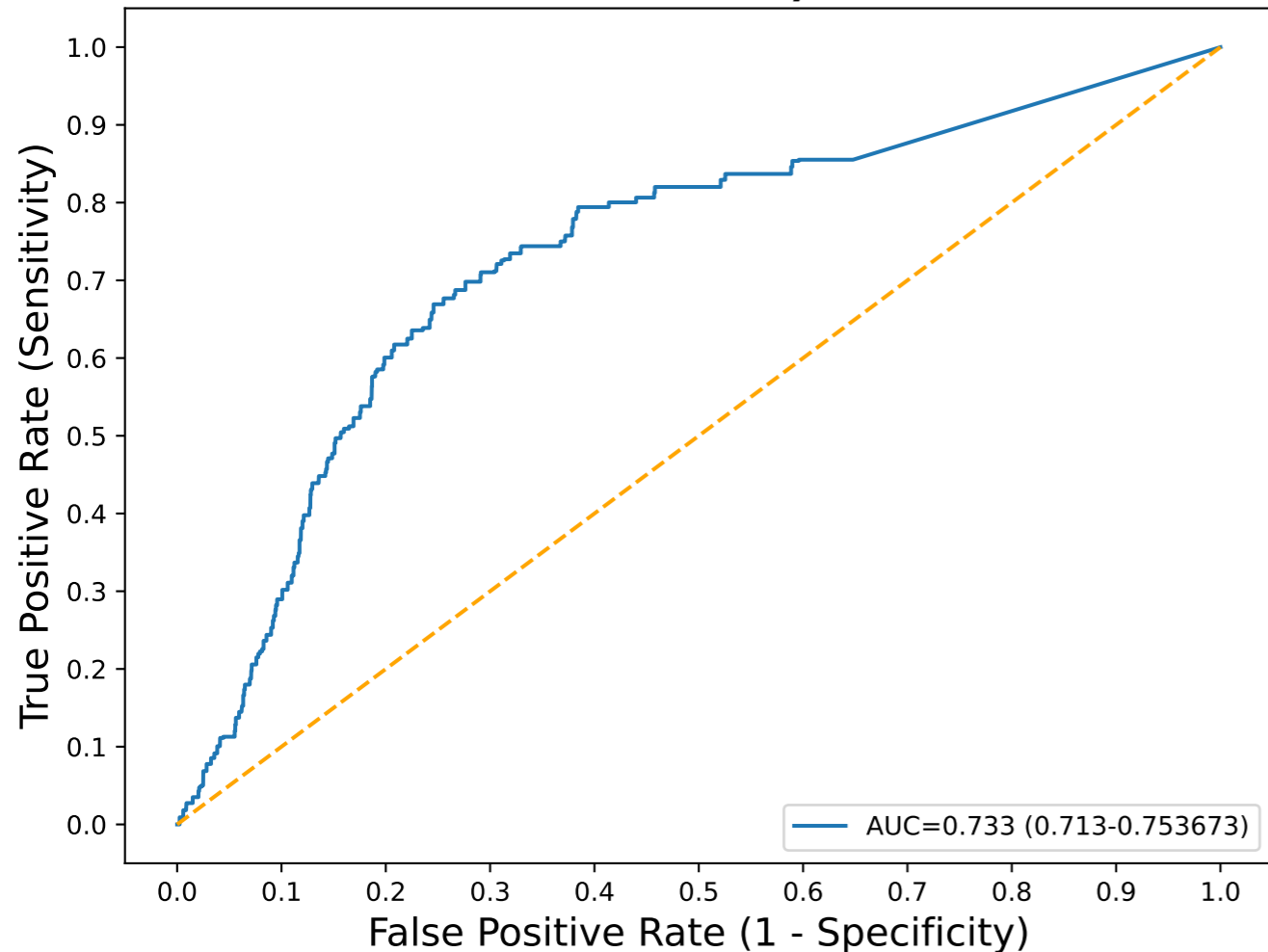

### ROC Curve Analysis RCA

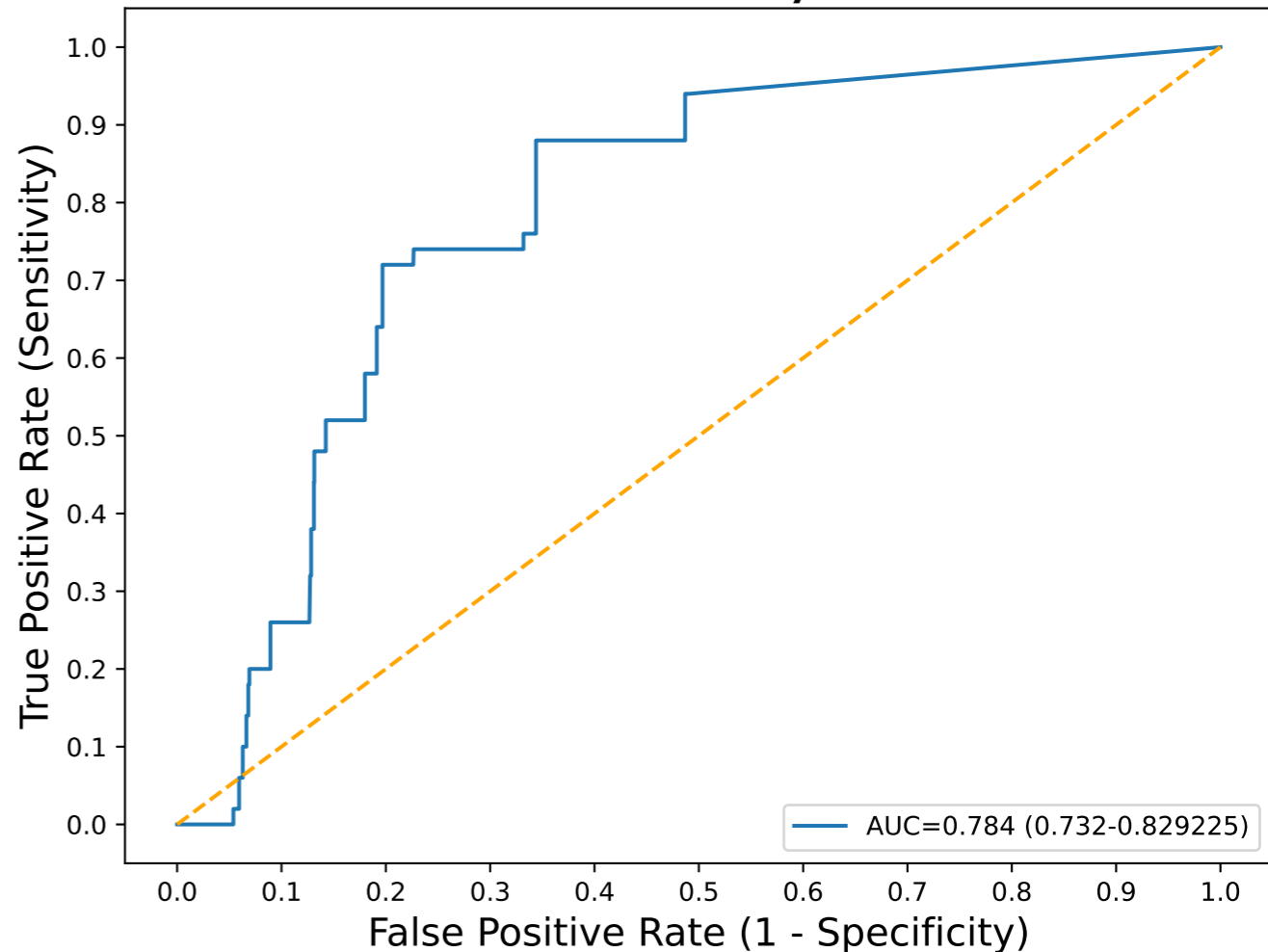

Supplement: Supplementary file 3 — Supplementary file3 (PDF 37 KB) [file 10554_2025_3324_MOESM3_ESM.pdf]

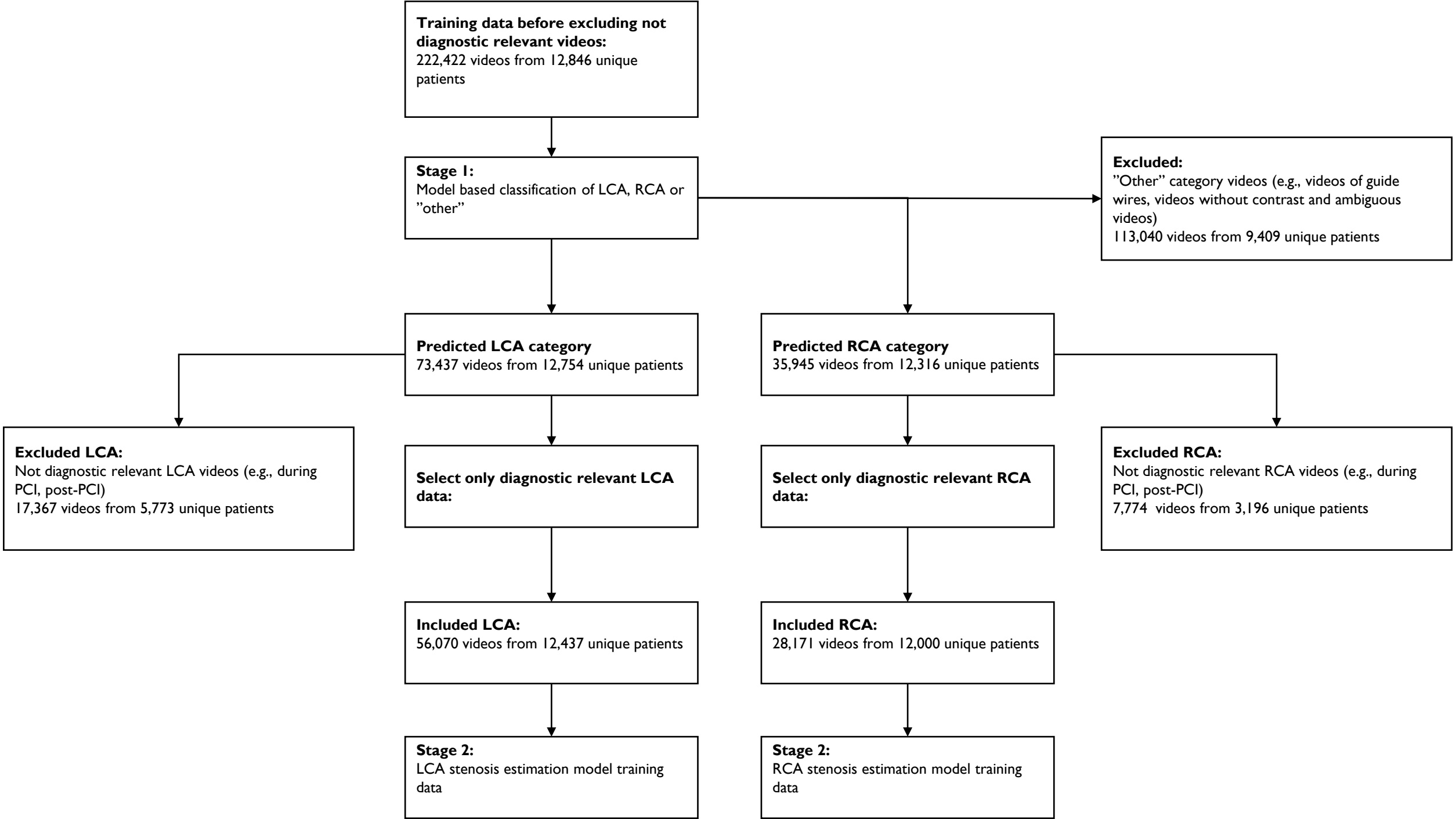

Supplement: Supplementary file 5 — Supplementary file5 (PDF 78 KB) [file 10554_2025_3324_MOESM5_ESM.pdf]

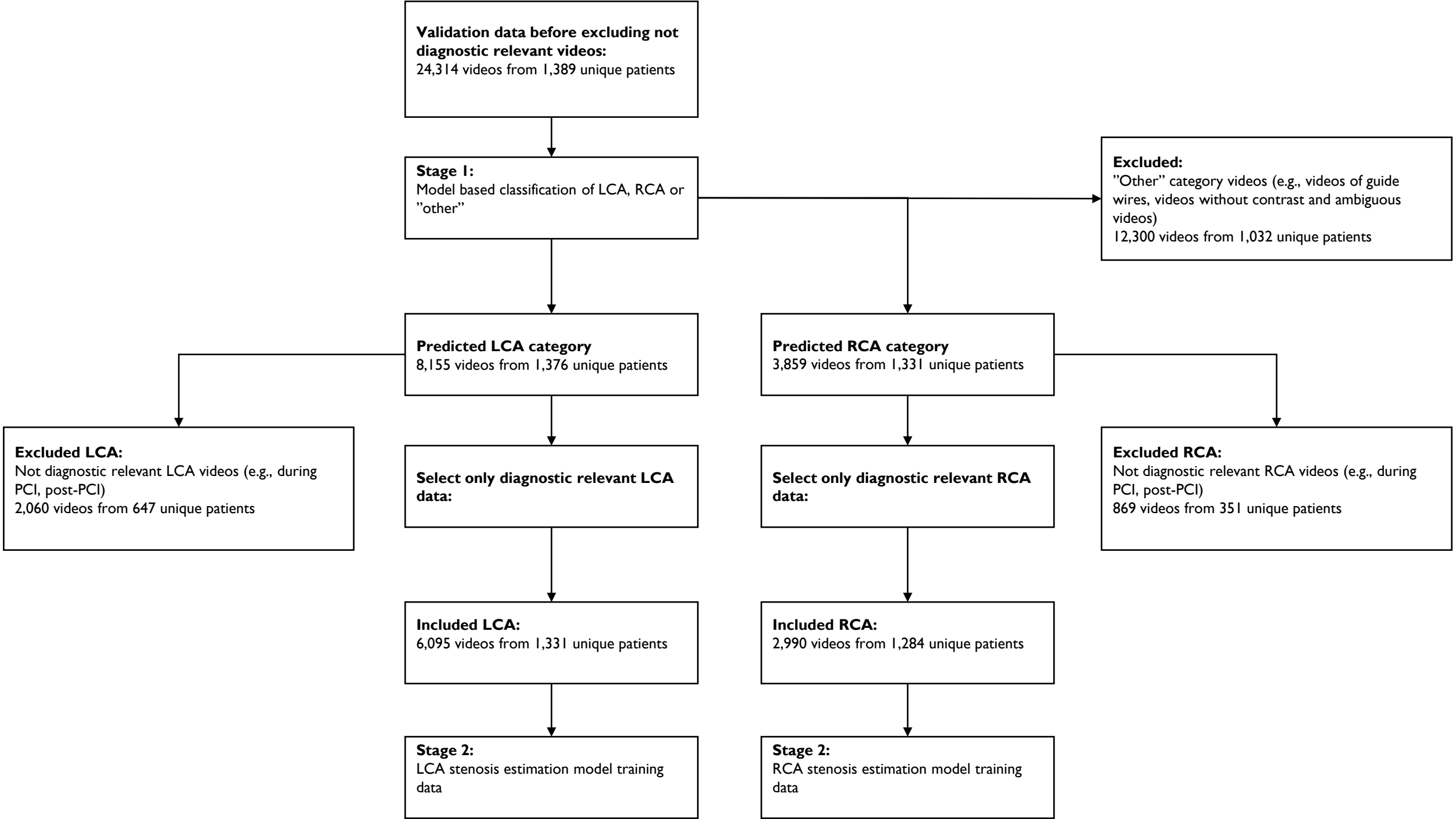

Supplement: Supplementary file 6 — Supplementary file6 (PDF 78 KB) [file 10554_2025_3324_MOESM6_ESM.pdf]

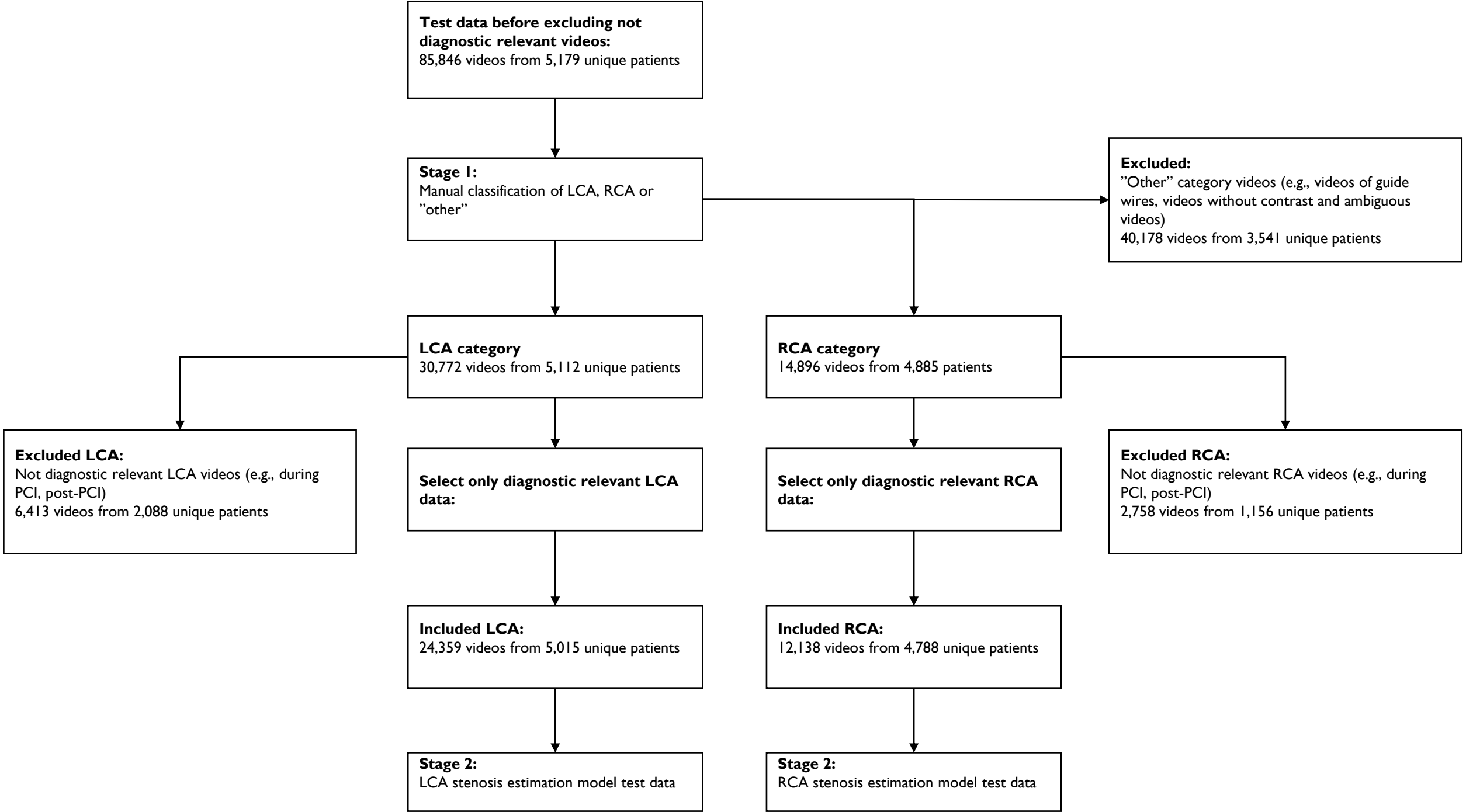

Supplement: Supplementary file 7 — Supplementary file7 (PDF 78 KB) [file 10554_2025_3324_MOESM7_ESM.pdf]

Confusion Matrix on test set

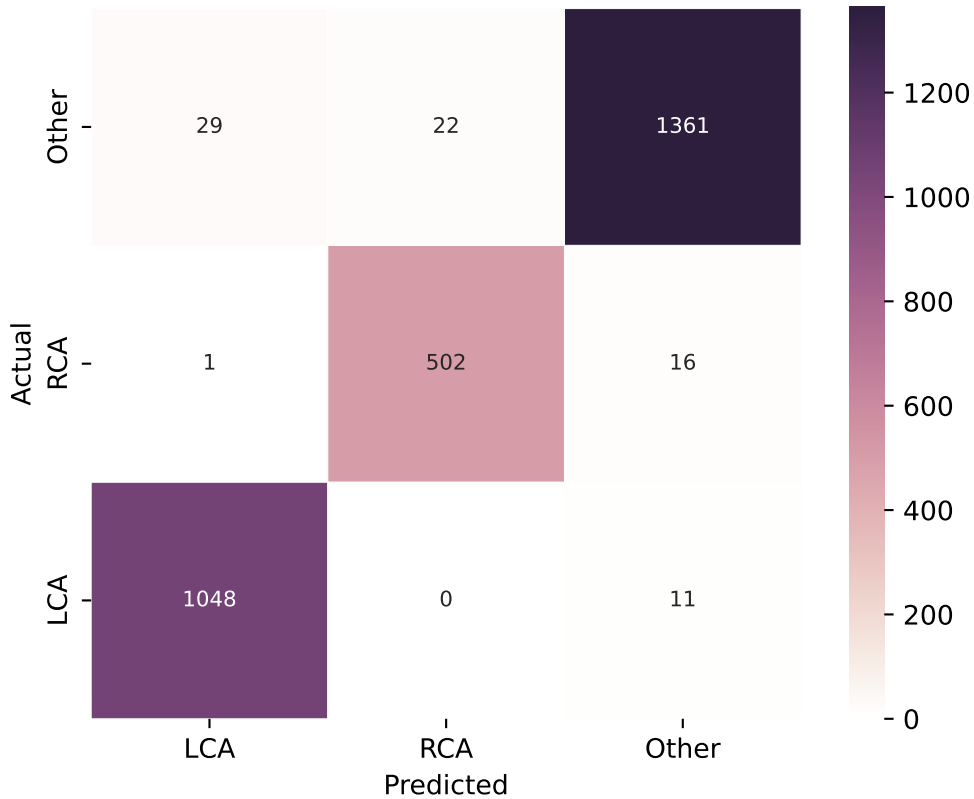

Supplement: Supplementary file 8 — Supplementary file8 (PDF 15 KB) [file 10554_2025_3324_MOESM8_ESM.pdf]

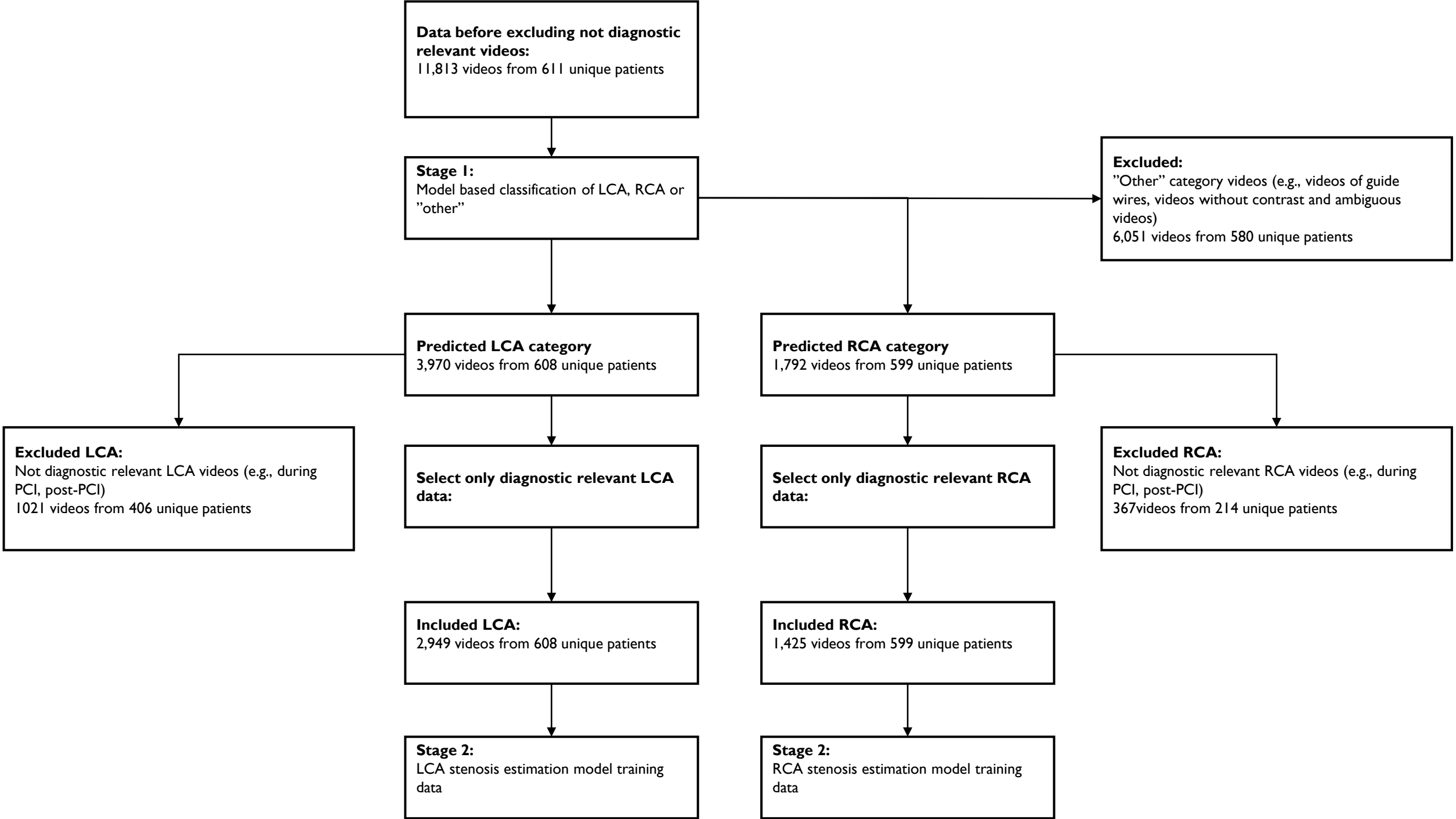

Supplement: Supplementary file 11 — Supplementary file11 (PDF 56 KB) [file 10554_2025_3324_MOESM11_ESM.pdf]

LCA

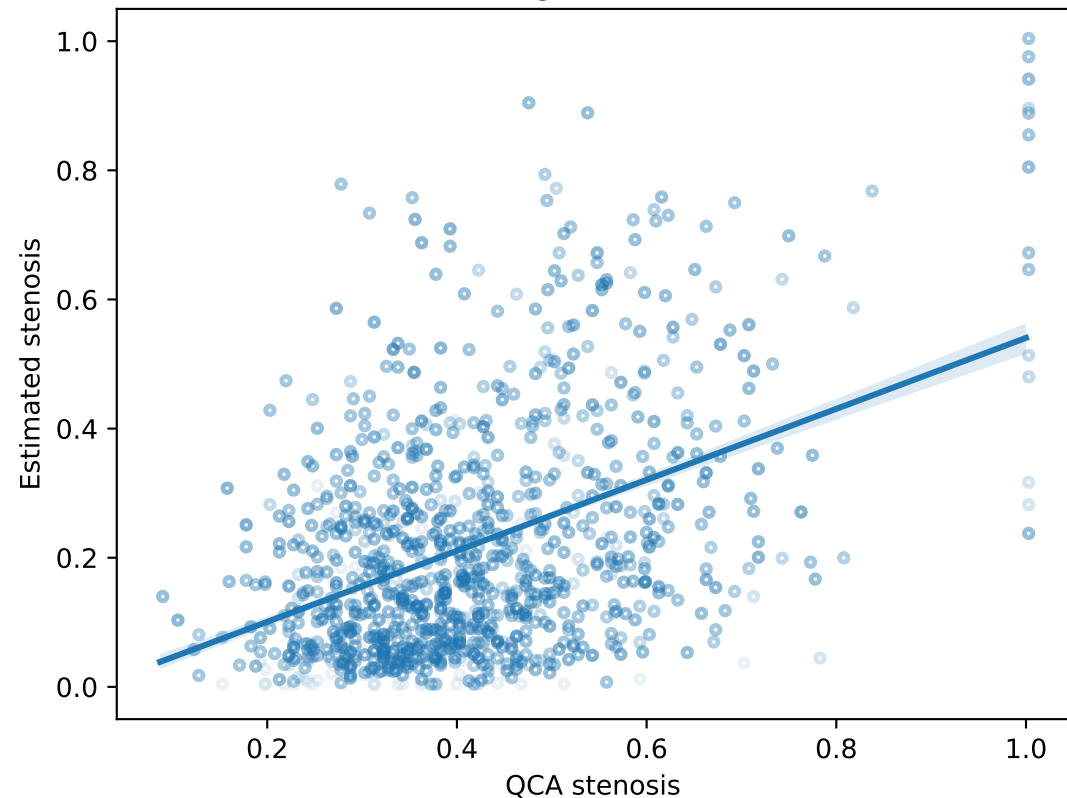

RCA

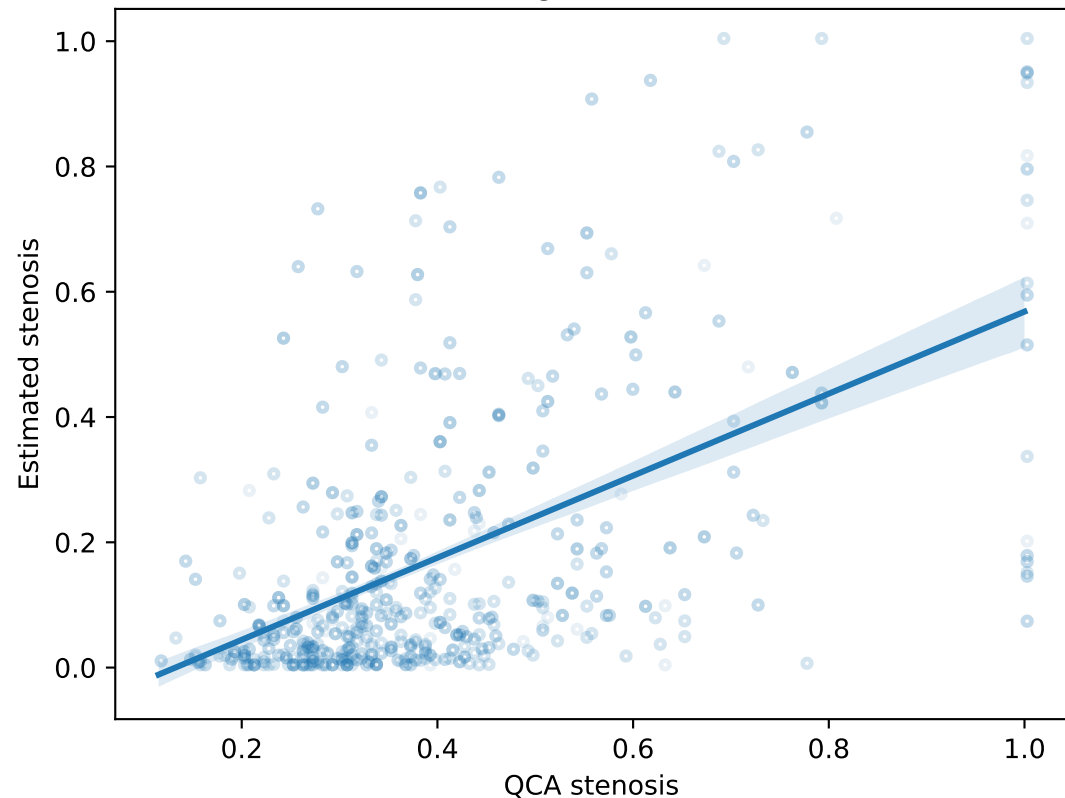

Supplement: Supplementary file 12 — Supplementary file12 (PDF 227 KB) [file 10554_2025_3324_MOESM12_ESM.pdf]
