# Supplementary material for: Automated stenosis estimation of coronary angiographies using end-to-end learning
Source: Int J Cardiovasc Imaging. 2025 Jan 9;41(3):441–52. doi: 10.1007/s10554-025-03324-x (PMC11880145; doi:10.1007/s10554-025-03324-x)
Supplement: Supplementary file 4 — Supplementary file4 (PDF 167 KB) [file 10554_2025_3324_MOESM4_ESM.pdf]

**Video**	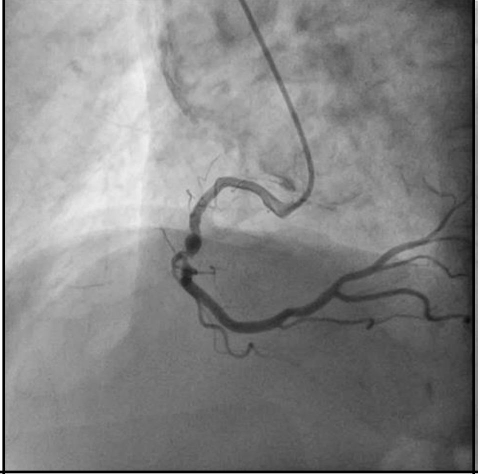	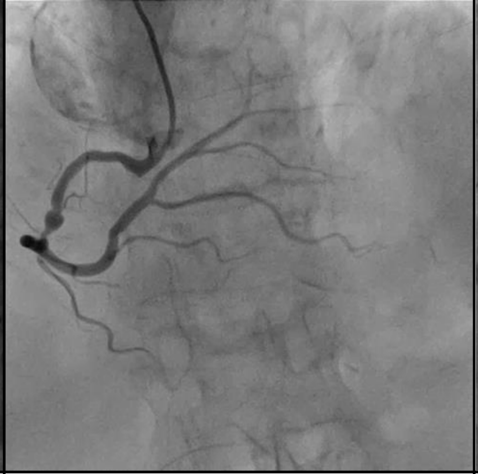	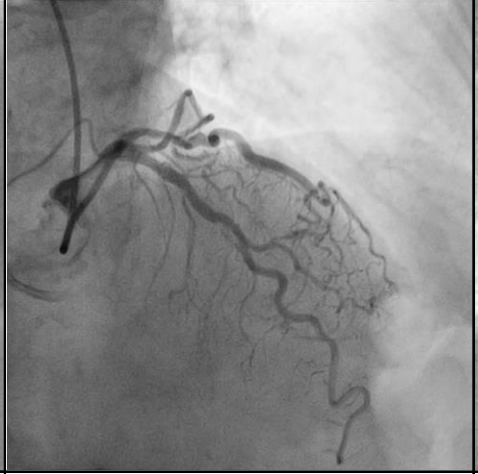	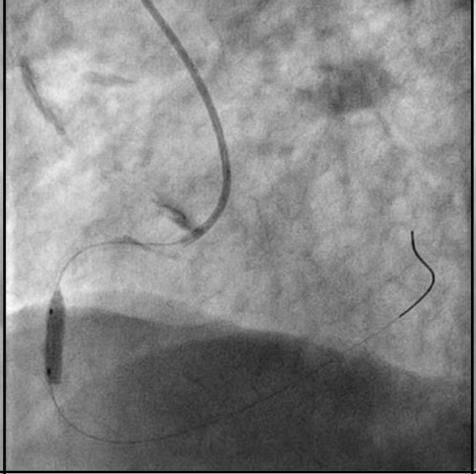	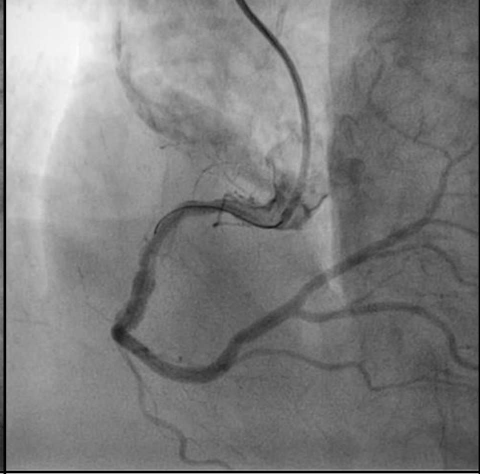
**Cine loop nr**	1	2	5	22	25
**Timestamp**	12:00:00	12:05:00	12:15:00	12:30:00	13:00:00
**Description**	Visual assessment of stenosis on RCA	Visual assessment of stenosis on RCA	Visual assessment of stenosis on LCA	PCI on RCA	Visualizing the outcome of the PCI on RCA
**Classified by model**	RCA	RCA	LCA	"Other"	RCA
**Select diagnostic relevant cine loops**	Include	Include	Include	Exclude	Exclude
