# Supplementary material for: Automated stenosis estimation of coronary angiographies using end-to-end learning
Source: Int J Cardiovasc Imaging. 2025 Jan 9;41(3):441–52. doi: 10.1007/s10554-025-03324-x (PMC11880145; doi:10.1007/s10554-025-03324-x)
Supplement: Supplementary file 9 — Supplementary file9 (PDF 186 KB) [file 10554_2025_3324_MOESM9_ESM.pdf]

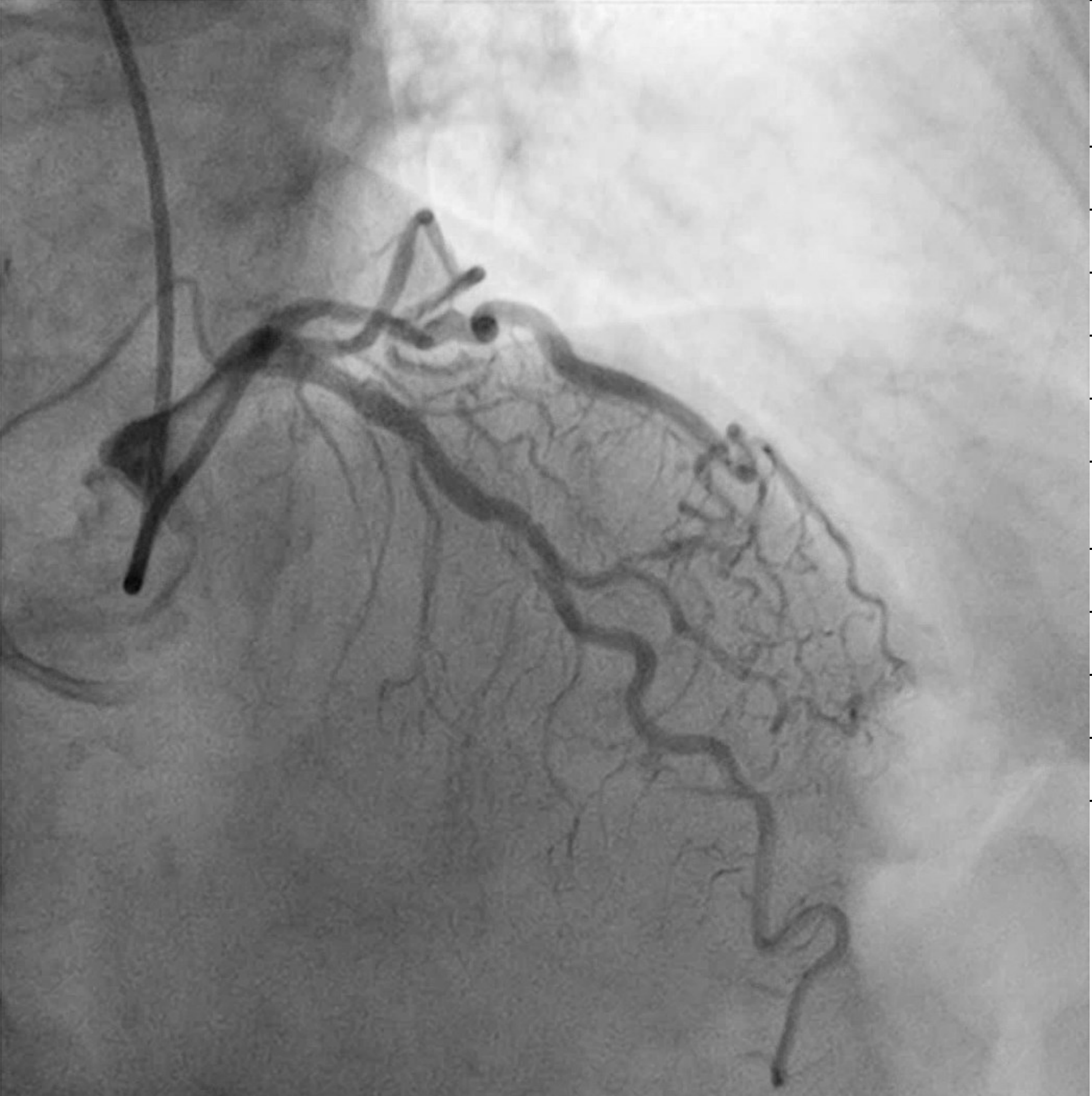

Segments	Estimated stenosis	Visual assessment
5 LM	0.0109	
6 Proximal LAD	0.0293	
7 Middle LAD	0.0839	
8 Distale LAD	0.0220	
9 Diagonal 1	0.0232	
10 Diagonal 2	0.0042	
11 Proximal LCX	0.0234	
12 Marginal 1	0.0123	
13 Middle LCX	0.033	
14 Marginal 2	0.0038	
15 Distale LCX	0.0098	
