# Supplementary material for: Automated stenosis estimation of coronary angiographies using end-to-end learning
Source: Int J Cardiovasc Imaging. 2025 Jan 9;41(3):441–52. doi: 10.1007/s10554-025-03324-x (PMC11880145; doi:10.1007/s10554-025-03324-x)
Supplement: Supplementary file 10 — Supplementary file10 (PDF 179 KB) [file 10554_2025_3324_MOESM10_ESM.pdf]

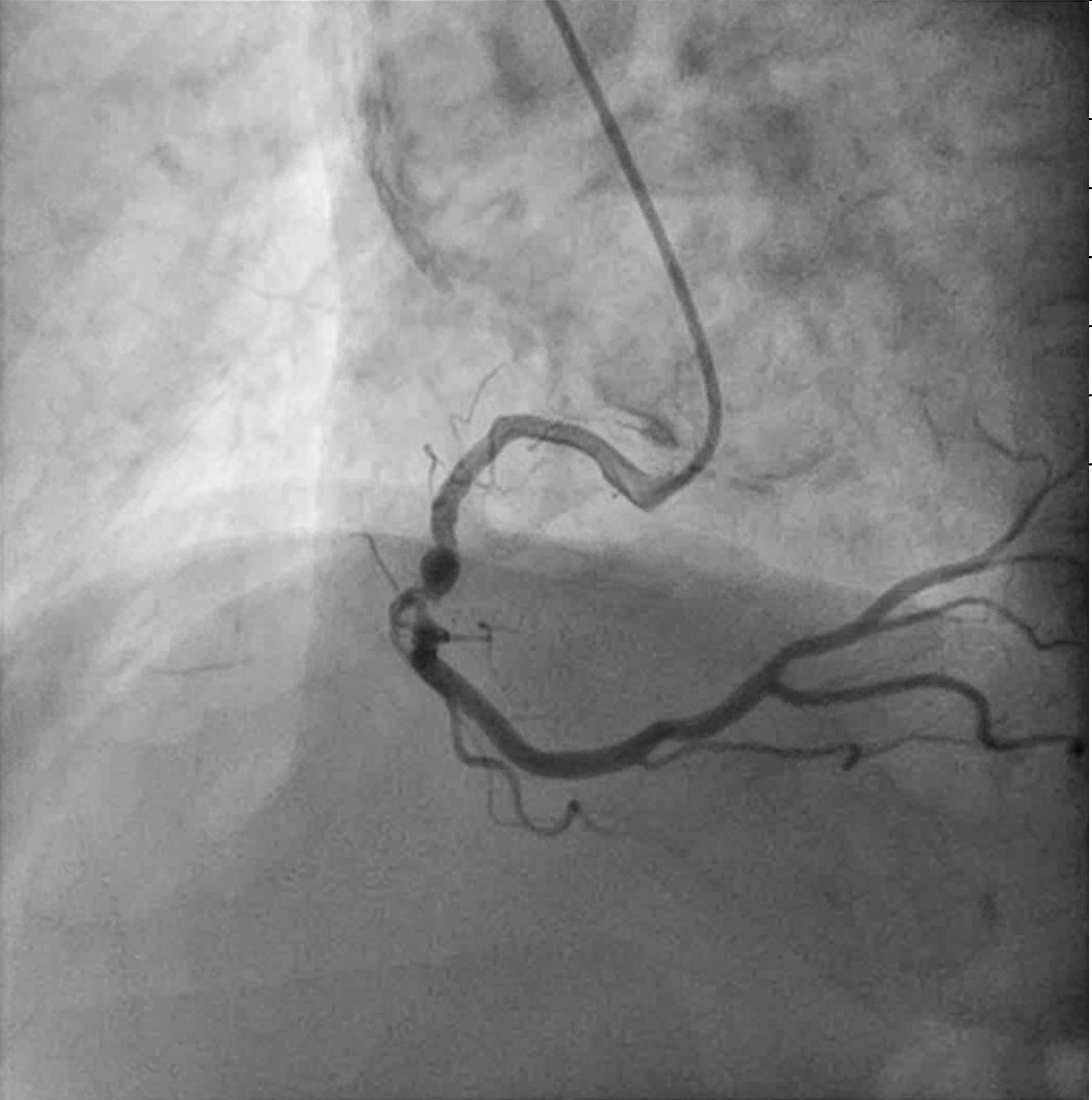

Segments	Estimated stenosis	Visual assessment
1 Proximal RCA	0.0075	
2 Middle RCA	0.7414	0.80
3 Distale RCA	0.0	
4 PDA	0.0068	
16 PLA RCA	0.0147	
